# The Feature Description of Formal Context Based on the Relationships among Concept Lattices

**DOI:** 10.3390/e23111397

**Published:** 2021-10-24

**Authors:** Ting Qian, Yongwei Yang, Xiaoli He

**Affiliations:** 1 College of Science, Xi’an Shiyou University, Xi’an 710065, China; hexl@xsyu.edu.cn; 2 School of Mathematics and Statistics, Anyang Normal University, Anyang 455000, China; yangyw@aynu.edu.cn

**Keywords:** formal context, concept lattice, the three-way decision, the three-way object oriented concept lattice, II-dual intersectable context

## Abstract

Three-way concept analysis (3WCA) is extended research of formal concept analysis (FCA) by combining three-way decision. The three-way object oriented concept lattice (OEOL) is one of the important data structures which integrates rough set, concept lattice and three-way decision in 3WCA. In the paper, we investigate the characteristics of formal context based on the isomorphic relationship among the kinds of concept lattices with OEOL. Firstly, II-dual intersectable attributes and II-dual intersectable context are proposed and the relationship between the type I-dual intersectable context(dual intersectable context) and the type II-dual intersectable context are studied. In addition, the relationship among the kinds of concept lattices with OEOL are studied when the formal context is both I-dual intersectable context and II-dual intersectable context. Finally, the inverse problems of the above conclusions are discussed and the following two conclusions are obtained: (1) the formal context is the type II-dual intersectable context, when the object oriented concept lattice and OEOL are isomorphic. (2) In addition, the formal context is the type I-dual intersectable context, when the concept lattice and OEOL are anti-isomorphic.

## 1. Introduction

In order to find an application carrier for lattice, FCA was proposed by Wille in 1982 [1,2]. It studies the hierarchical structures (that is, concept lattice) which is induced by a binary relation of objects and attributes. In recent years, FCA becomes an helpfull tool for data processing and knowledge discovery [3,4,5,6,7,8,9,10]. For adapting to different data analysis, Wang et al. studied the AFS concept lattice [11]. Ma et al. proposed the variable threshold concept lattice [12]. Guo et al. provided the power concept lattice [13]. Li et al. gave the approximate concept lattice [14] based on incomplete data. Düntsch and Gediga [15] and Yao [16] proposed the property oriented concept the lattice and object oriented concept lattice, respectively, and so on.

Recently, an outline of three-way decision was proposed [17]. Conflict analysis and FCA has been widely developed based on three-way decision. Three-way conflict analysis was proposed and studied [18,19,20]. In addition, 3WCA was firstly introduced by Qi, Wei and Yao in 2014 [21]. Then, 3WCA has become one of the helpful research problems. Firstly, Qi et al. gave the connections between the three-way concept lattice and classical concept lattice [22]. In terms of lattice building, Qian et al. proposed the method based formal context from the perspective of context [23]. Later, Yang et al. in 2020 proposed the method based on the composite of classical lattices from the perspective of lattice [24]. In terms of attribute reduction, Ren et al studies the reduction theory of three-way concept lattices from the perspectives of preserving intersection (Union) irreducible elements, preserving granular and preserving lattice structure, respectively [25].

In addition, inspired by 3WCA, some new three-way concept lattice models are proposed. Firstly, OEOL was proposed by Wei and Qian based on the ideal of locally completely (no-) possessing [26]. Later, Qian et al. discussed them more comprehensively and detailedly in [27]. Li et al. put forward three-way cognitive concepts based on cognitive theory and studied them through multi granularity method [28]. Conflict analysis model was proposed based on 3WCA by Zhi et al. [29]. Mao and Cheng proposed the three-way rough semiconcept in 2021 [30]. Meanwhile, intuitionistic fuzzy three-way formal concept analysis were studied by using attribute correlation degree in 2021 [31].

However, 3WCA are much more complex than FCA because of the large amount of information the three-way concept lattices carried. So, this brings us a lot of trouble in dealing with them. In addition, FCA has improved maturely. Therefore, it is a good idea to deal with the problems of 3WCA by using the related methods of FCA. Along the above research ideas, Yang et al. used the composite of classical lattices to construct the three-way concept lattices [24]. Long et al. studied attribute-induced three-way concept lattice in incomplete fuzzy formal context by double threshold construction method [32]. Chen et al. used unlabelled text mining methods to study object-induced three-way concept lattice [33]. From the perspective of formal context characteristics, Qian et al. gave a kind of attribute characteristics, that is $G\backslash a^{*}=\cap \left(a_{j}^{*}\right)$, which is called the dual intersectable attribute (later, we call it the type I-dual intersectable attribute. The corresponding formal context is called type I-dual intersectable formal context). Furthermore, they studied the isomorphic relation between concept lattice and three-way concept lattices based on the dual intersectable formal context [34].

The literature [34] tells us that $G\backslash a^{*}=\cap \left(a_{j}^{*}\right)$ means original information describes supplementary information. Inspired by it, we consider another attribute feature by using supplementary information to describe original information and then we investigate the isomorphism among concept lattices, especially the relationship between concept lattice and OEOL in this paper.

The rest of the paper is organized as follows. Section 2 reviewes some notions briefly. In Section 3, we propose the type II-dual intersectable formal context and investigate the relations among some concept lattices including concept lattice, the object oriented concept lattice and OEOL when the formal context is the type II-dual intersectable context. In Section 4, the inverse proposition of related conclusions in Section 3 are given. In addition, several theorems and examples are given. Finally, the summary is given by using a diagram in Section 5.

## 2. Preliminaries

### 2.1. Concept Lattice

Firstly, some basic definitions are given.

**Definition** **1.** *[2] Let G and M be two finite sets and I be a binary relation between G and M, we call (G,M,I) a formal context. In addition, g∈G and m∈M are called the object and the attribute, respectively. (g,m)∈I means that the object g has the attribute m.*

*A pair of dual operators ∗ for any X⊆G and A⊆M and the definitions of formal concept and concept lattice are given as follows.*

$$X^{*}=\left\{m\in M\right|gIm\;for\;all\;g\in X\},\;A^{*}=\left\{g\in G\right|gIm\;for\;all\;m\in A\}.$$

*(X,A), X and A are, respectively, called a formal concept, an extent and an intent when $X^{*}=A$ and $A^{*}=X$. If X={m} and A={g}, then ${\left\{m\right\}}^{*}$ and ${\left\{g\right\}}^{*}$ are abbreviated to $m^{*}$ and $g^{*}$, respctively.*

*If $\forall g\in G,g^{*}\neq \emptyset ,g^{*}\neq M$, and $\forall m\in M,m^{*}\neq \emptyset ,m^{*}\neq G$, we call (G,M,I) the canonical context.*

*If $\forall g,h\in G,g^{*}\neq h^{*}$, and $\forall m,n\in M,m^{*}\neq n^{*}$, we call (G,M,I) the clarified context.*

*The set of all formal concepts is called the concept lattice denoted by L(G,M,I). For any (X,A),(Y,B)∈L(G,M,I), the partial order is defined by:*

(X,A)⩽(Y,B)⇔X⊆Y(⇔A⊇B).

*And it is easy to prove it is a complete lattice with the above partial order.*

**Remark** **1.** *(1) Extent (intent) is still extent (intent) after the intersection of sets. And the properties of operators are shown in the literature [2]; (2) Every set in a lattice except G, M and *∅* is also expressed directly by listing its elements.*

**Figure 1:**
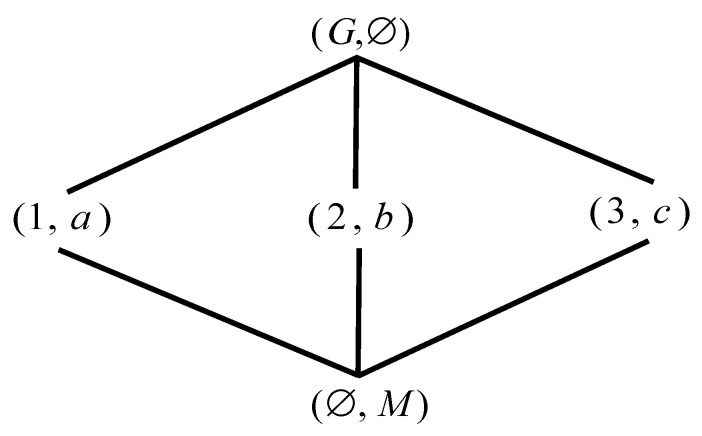
L(G,M,I) of Table 1.

**Example** **1.** *Let G={1,2,3} and M={a,b,c}, I is represented in Table 1. We get a formal context. According to Definition 1, The concepts are calculated easily, and then the correspoding concept lattice is represented by Figure 1.*

### 2.2. The Object Oriented Concept Lattice

The object oriented concept lattice was proposed by Yao in [16]. In addition, it is recalled as follows.

**Definition** **2.** *[16] Let a formal context be (G,M,I). For any X⊆G and A⊆M, a pair of operators □:P(G)→P(M) and ◊:P(M)→P(G) as follows.*

$$X^{□}=\left\{m\in M|m^{*}\subseteq X\right\},\;A^{◊}=\{g\in G|g^{*}\cap A\neq \emptyset \}.$$

*(X,A) is called an object oriented concept. X and A are, respectively, called the extent and the intent of (X,A) when $X^{□}=A$ and $A^{◊}=X$. The set of all object oriented concepts form a complete lattice which is called the object oriented concept lattice and is denoted by $L_{o}\left(G,M,I\right)$. The partial order on it is defined as follows:*

(X,A)⩽(Y,B)⇔X⊆Y(⇔A⊆B).

**Remark** **2.** *Extent is still extent after the union of sets. And the properties of operators are shown in the literature [16].*

**Figure 2:**
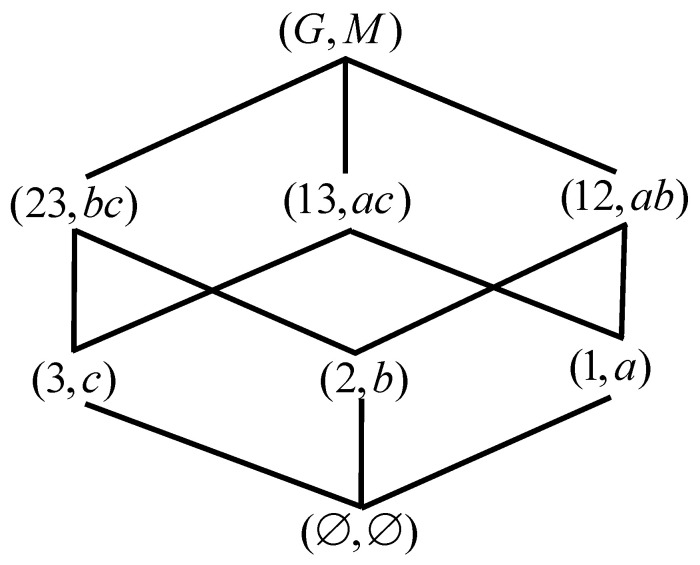
$L_{o}\left(G,M,I\right)$ of Table 1.

**Example** **2.** *$L_{o}\left(G,M,I\right)$ of Table 1 is shown in Figure 2 according to Definition 2.*

### 2.3. The Three-Way Object Oriented Concept Lattice

Inspired by 3WCA, Wei and Qian [26,27] proposed OEPL and OEOL. The relevant definitions are as follows. Firstly, the negative operators of ∗ are recalled.

**Definition** **3.** *[21,22] Let a formal context be (G,M,I) and $I^{c}=\left(G\times M\right)\backslash I$. For X⊆G,A⊆M, another pair of operators, $\bar{*}:P\left(G\right)\to P\left(M\right)$ and $\bar{*}:P\left(M\right)\to P\left(G\right),$ are called negative operators, as follows. For X⊆G and A⊆M,*

$$X^{\bar{*}}=\left\{m\in M|\forall x\in X\left(xI^{c}m\right)\right\},\;A^{\bar{*}}=\{g\in G|\forall a\in A\left(gI^{c}a\right)\}.$$

Based on $\bar{*}$, the following pair of new negative operators of □ and *◊* are given by using the semantics of locally completely (no-) possessing.

**Definition** **4.** *[26] Let a formal context be (G,M,I). For X⊆G and A⊆M, new negative operators are defined as follows: $\bar{□}:P\left(G\right)\to P\left(M\right)$ and $\bar{◊}:P\left(M\right)\to P\left(G\right),$ for X⊆G and A⊆M, $X^{\bar{□}}=\left\{m\in M|m^{\bar{*}}\subseteq X\right\},$ $A^{\bar{◊}}=\left\{g\in G|g^{\bar{*}}\cap A\neq \emptyset \right\}.$*

Wei and Qian gave OEO-operators, OEO-concept and OEOL by combing the operators □, *◊*, $\bar{□}$ and $\bar{◊}$ together.

**Definition** **5.** *[26] Let a formal context be (G,M,I). For X⊆G and A,B⊆M, a new pair of operators(OEO operators), ▹:P(G)→DP(M) and ◃:DP(M)→P(G), are defined by $X^{▹}=\left(X^{□},X^{\bar{□}}\right)$ and ${\left(A,B\right)}^{◃}=A^{◊}\cup B^{\bar{◊}}$, respectively.*

*(X,(A,B)), X and (A,B) are, respectively, called a three-way object oriented concept (OEO-concept), an extent and an intent, if and only if $X={\left(A,B\right)}^{◃}$ and $X^{▹}=\left(A,B\right)$. The family of all OEO-concepts of (G,M,I) is denoted as OEOL(G,M,I), and (OEOL(G,M,I),≤) is abbreviated as OEOL, where ≤ is (X,(A,B))≤(Y,(C,D))⇔X⊆Y⇔(A,B)⊆(C,D).*

**Figure 3:**
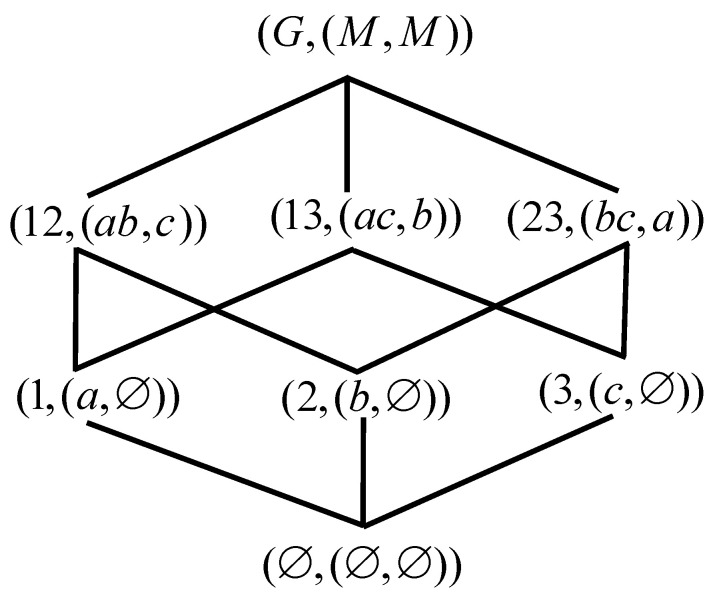
OEOL(G,M,I) of Table 1.

**Example** **3.** *OEOL(G,M,I) of Table 1 is shown in Figure 3 according to Definition 5.*

## 3. The Relations between Kinds of Concept Lattices and OEOL

In this section, the relations between kinds of concept lattice and OEOL based on some context are explored firstly.

For the convenience of description, we give the following symbols:

The set consisting of all extents of the formal concepts, the set of all extents of the object oriented concepts and the family of all extents of the three-way object oriented conceptsis are, respectively, denoted by $L_{E}\left(G,M,I\right)$, $L_{oE}\left(G,M,I\right)$ and $OEOL_{E}\left(G,M,I\right)$.

### 3.1. The Type II-Dual Intersection Formal Context

In fact, Qian et al. studied the isomorphic relation between concept lattice and three-way concept lattices. In addition, then they proposed the dual intersectable context [34]. Similarly, we propose another form of dual feature of attributes in this subsection. In order to distinguish, we call the original dual feature of attributes as type I.

**Definition** **6.** *Let a formal context be (G,M,I), a∈M. If there are some $a_{j}\in M$ which satisfies $a^{*}=\cap \left(G\backslash a_{j}^{*}\right)$, then a is called a type II-dual intersection attribute.*

The attribute features described in Definition 6 means that original information are described supplementary information. Let’s explain it with the following example.

**Example** **4.** A formal context is represented in Table 2. By definition of *, we can compute that $a^{*}=\left\{2\right\}$$G\backslash b^{*}=\left\{1,2\right\}$, $G\backslash c^{*}=\left\{2,3\right\}$, so we get $a^{*}=\left(G\backslash b^{*}\right)\cap \left(G\backslash c^{*}\right)$. It’s easy for us to verify that a is a type II-dual intersection attribute by Definition 6.

Next, we describe the features of formal context based on the above attribute features. In addition, we explain it by examples.

**Definition** **7.** *Let a formal context be (G,M,I). If a is type II-dual intersection attribute for any a∈M, then (G,M,I) is called a attribute-induced type II-dual intersection formal context.*

**Example** **5.** *We can calculate the following results in turn from Table 2.*

*$a^{*}=\left\{2\right\}$, $G\backslash b^{*}=\left\{1,2\right\}$, $G\backslash c^{*}=\left\{2,3\right\}$, so we get $a^{*}=\left(G\backslash b^{*}\right)\cap (G\backslash c^{*}$).*

*$b^{*}=\left\{3\right\}$, $G\backslash a^{*}=\left\{1,3\right\}$, $G\backslash c^{*}=\left\{2,3\right\}$, so we get $b^{*}=\left(G\backslash a^{*}\right)\cap (G\backslash c^{*}$).*

*$c^{*}=\left\{1\right\}$, $G\backslash a^{*}=\left\{1,3\right\}$, $G\backslash b^{*}=\left\{1,2\right\}$, so we get $c^{*}=\left(G\backslash a^{*}\right)\cap (G\backslash b^{*}$).*

*$d^{*}=\left\{2,3\right\}$, $G\backslash c^{*}=\left\{2,3\right\}$, so we get $d^{*}=G\backslash c^{*}$.*

*$e^{*}=\left\{1\right\}$, $G\backslash d^{*}=\left\{1\right\}$, so we get $e^{*}=G\backslash d^{*}$.*

*It’s easy for us to verify (G,M,I) is a attribute-induced type II-dual intersection formal context by Definition 7.*

Let’s use a theorem to illustrate the relationship between type I-dual intersection formal context and type II-dual intersection formal context.

**Theorem** **1.** *Let (G,M,I) be a formal context. (G,M,I) is a type II-dual intersection formal context if and only if $\left(G,M,I^{c}\right)$ is a type I-dual intersection formal context, where $I^{c}=\left(G\times M\right)\backslash I$.*

**Proof.**  Since $X^{\bar{*}}=\left\{m\in M|\forall x\in X\left(\neg \left(xIm\right)\right)\right\}=\left\{m\in M|\forall x\in X\left(xI^{c}m\right)\right\},$ we get ${\left\{a\right\}}^{\bar{*}}=\left\{m\in M|\forall a\in \left\{a\right\}\left(\neg \left(aIm\right)\right)\right\}=\left\{m\in M|\forall a\in \left\{a\right\}\left(aI^{c}m\right)\right\}=\left\{m\in M|aI^{c}m\right\}=G\backslash a^{*}$. In the same way, $a^{*}=G\backslash a^{\bar{*}}$. Therefore, when (G,M,I) is a type II-dual intersection formal context, we obtain $a^{*}=\cap \left(G\backslash a_{j}^{*}\right)$ for any *a* by Definition 7. And then $a^{*}=\cap \left(G\backslash a_{j}^{*}\right)$ is equivalent to $G\backslash a^{\bar{*}}=\cap \left(a_{j}^{\bar{*}}\right)$. So we get $\left(G,M,I^{c}\right)$ is a type I-dual intersection formal context. vice versa. □

By the proof of Theorem 1, we can easily draw the relationship between type I and type II: (1) *a* is a type II-dual intersection attribute of (G,M,I) if and only if *a* is a type I-dual intersection attribute of $\left(G,M,I^{c}\right)$; (2) (G,M,I) is a type II-dual intersection formal context if and only if $\left(G,M,I^{c}\right)$ is a type I-dual intersection formal context.

### 3.2. The Relationship between Kinds of Concept Lattices and OEOL Based on the Type II-Dual Intersection Context

In the subsection, we will study the relationship among kinds of concept lattices based on the type II-dual intersection context. Firstly, the conclusions about the object oriented concept lattices based on the type II-dual intersection context are discussed.

**Theorem** **2.** *Let a formal context be (G,M,I). If (G,M,I) is a type II-dual intersection formal context, then $L_{oE}\left(G,M,I^{c}\right)\subseteq L_{oE}\left(G,M,I\right)$.*

**Proof.**  For any a∈M, we get $a^{\bar{◊}}\in L_{oE}\left(G,M,I^{c}\right)$ by the property of operators $\bar{◊}$. And $a^{\bar{◊}}=\left\{g\in G|g^{\bar{*}}\cap \left\{a\right\}\neq \emptyset \right\}=\left\{g\in G|a\in g^{\bar{*}}\right\}=\left\{g\in G|gIa\right\}=a^{\bar{*}}=G\backslash a^{*}$. Similarly, we can get $a^{◊}=a^{*}$. Since (G,M,I) is a type II-dual intersection formal context, we can get $a^{*}=\cap \left(G\backslash a_{j}^{*}\right)$. Thus, we can get $G\backslash a^{*}=\cup \left(a_{j}^{*}\right)$ by De Morgan’s law. That is, $a^{\bar{◊}}=\cup \left(a_{j}^{◊}\right)$. In addition, we know $a_{j}^{◊}\in L_{oE}\left(G,M,I\right)$. So, we know $a^{\bar{◊}}\in L_{oE}\left(G,M,I\right)$ by the property of operators *◊*. For any $\left(X,A\right)\in L_{o}\left(G,M,I^{c}\right)$, we can get $X={⋃}_{a_{j}\in A}a_{j}^{\bar{◊}}$. Thus, $X\in L_{oE}\left(G,M,I\right)$ by the property of operators *◊*. Therefore, $L_{oE}\left(G,M,I^{c}\right)\subseteq L_{oE}\left(G,M,I\right)$. □

Combing Theorem 1 and Theorem 2, we can easily obtain the following conclusion.

**Theorem** **3.** *Let (G,M,I) be a formal context. If (G,M,I) is both a type I-dual intersection formal context and a type II-dual intersection formal context, then $L_{oE}\left(G,M,I\right)=L_{oE}\left(G,M,I^{c}\right)$.*

**Theorem** **4.** *Let a formal context be (G,M,I). If (G,M,I) is a type II-dual intersection formal context, then $L_{o}\left(G,M,I\right)$ and OEOL(G,M,I) are isomorphic.*

**Proof.**  For any (X,(A,B))∈OEOL(G,M,I), we get $A=X^{□},B=X^{\bar{□}}$ and $X=A^{◊}\cup B^{\bar{◊}}$ by Definition 5. Since (G,M,I) is a type II-dual intersection formal context, we can obtain $L_{oE}\left(G,M,I^{c}\right)\subseteq L_{oE}\left(G,M,I\right)$ by Theorem 2. And then $B^{\bar{◊}}\in L_{oE}\left(G,M,I\right)$. Thus, $X=A^{◊}\cup B^{\bar{◊}}\in L_{oE}\left(G,M,I\right)$. Therefore, $OEOL_{E}\left(G,M,I\right)\subseteq L_{oE}\left(G,M,I\right)$. In addition, for any $\left(X,A\right)\in L_{o}\left(G,M,I\right)$, we can easily get $\left(X,\left(X^{□},X^{\bar{□}}\right)\right)\in OEOL\left(G,M,I\right)$ by the properties of □ and *◊*. That is, $L_{oE}\left(G,M,I\right)\subseteq OEOL_{E}\left(G,M,I\right)$. In summary, $L_{oE}\left(G,M,I\right)=OEOL_{E}\left(G,M,I\right)$. Therefore, $L_{o}\left(G,M,I\right)$ and OEOL(G,M,I) are isomorphic. □

Next, the following conclusions about concept lattices based on the type II-dual intersection context are drawn.

**Theorem** **5.** *Let a formal context be (G,M,I). If (G,M,I) is both a type I-dual intersection formal context and a type II-dual intersection formal context, then $L_{E}\left(G,M,I\right)=L_{E}\left(G,M,I^{c}\right)$.*

**Proof.**  Since (G,M,I) is a type I-dual intersection formal context, we get that there are some $a_{j}\in M$ which satisfies $G\backslash a^{*}=\cap \left(a_{j}^{*}\right)$, for any a∈M. That is $a^{\bar{*}}=\cap \left(a_{j}^{*}\right)$, for any a∈M. Thus, $a^{\bar{*}}\in L_{E}\left(G,M,I\right)$ by the property of operators. Therefore, $L_{E}\left(G,M,I^{c}\right)\subseteq L_{E}\left(G,M,I\right)$. Similarly, since (G,M,I) is also a type II-dual intersection formal context, we get $L_{E}\left(G,M,I\right)\subseteq L_{E}\left(G,M,I^{c}\right)$. So $L_{E}\left(G,M,I\right)=L_{E}\left(G,M,I^{c}\right)$. □

Through Theorem 5, we can easily establish the following conclusion by constructing isomorphic mapping.

**Theorem** **6.** *Let a formal context be (G,M,I). If (G,M,I) is both a type I-dual intersection formal context and a type II-dual intersection formal context, then L(G,M,I) and $L(G,M,I^{c})$ are isomorphic.*

**Proof.**  Let $\gamma :L\left(G,M,I\right)\to L\left(G,M,I^{c}\right)$, for any (X,A)∈L(G,M,I), $\gamma \left(X,A\right)=\left(X,X^{\bar{*}}\right)$. So we can easily prove that it’s an isomorphic mapping. Thus, we obtain L(G,M,I) and $L(G,M,I^{c})$ are isomorphic. □

Let’s illustrate that the inverse proposition of Theorem 6 does not hold by an example.

**Figure 4:**
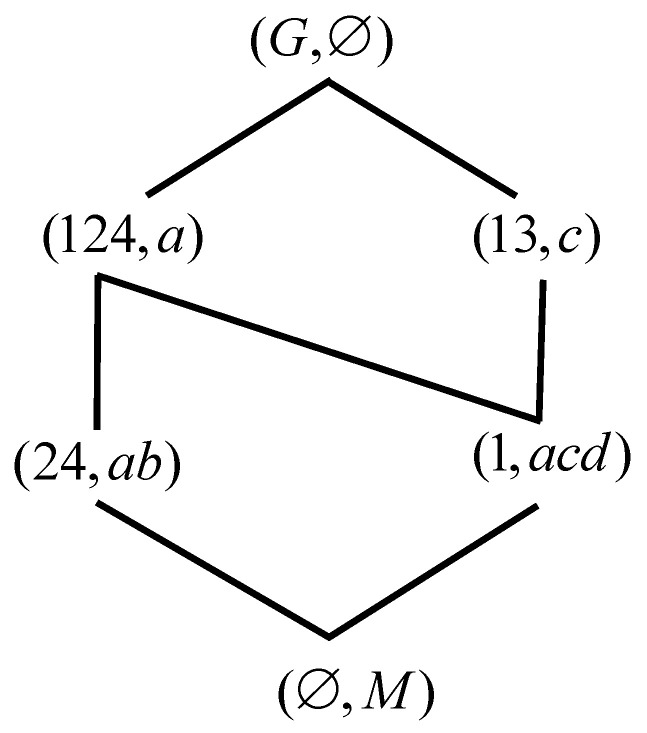
L(G,M,I) of Table 3.

**Figure 5:**
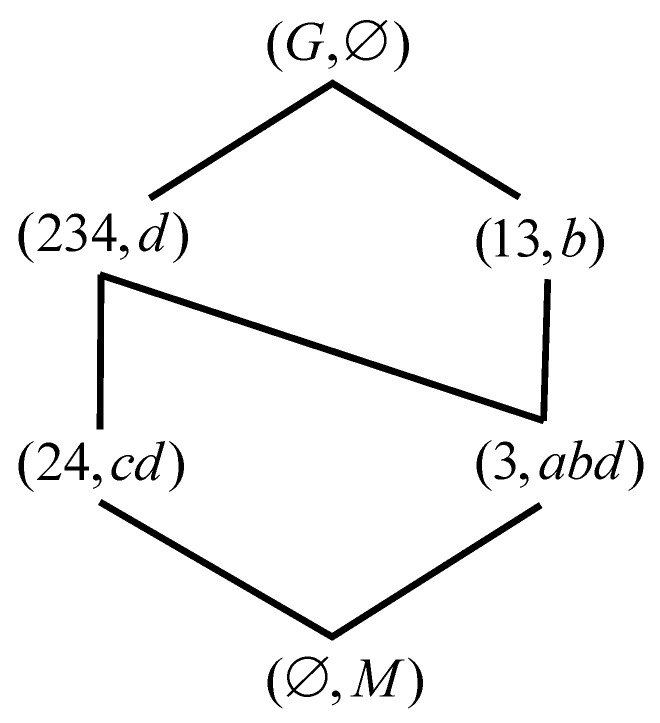
$L(G,M,I^{c})$ of Table 3.

**Example** **6.** *A formal context (G,M,I) is shown in Table 3. Figure 4 and Figure 5 are L(G,M,I) and $L(G,M,I^{c})$, respectively. We can obtain $a^{*}=\left\{1,2,4\right\}$, $b^{*}=\left\{2,4\right\}$, $c^{*}=\left\{1,3\right\}$, $d^{*}=\left\{1\right\}$, $G\backslash d^{*}=\left\{2,3,4\right\}$ from Table 3. In addition, it’s easy for us to verify d is not a type type I-dual intersection attribute, Thus, the formal context of Table 3 is not a type I-dual intersection formal context. But we can get L(G,M,I) and $L(G,M,I^{c})$ are isomorphic from Figure 4 and Figure 5. In fact, we can summarily compute $G\backslash a^{*}=\left\{1\right\}$, $G\backslash b^{*}=\left\{1,3\right\}$, $G\backslash c^{*}=\left\{2,4\right\}$, $G\backslash d^{*}=\left\{2,3,4\right\}$, $a^{*}=\left\{1,2,4\right\}$ from Table 3. And it’s easy for us to verify a is not a type II-dual intersection attribute by Definition 6. Thus, the formal context of Table 3 is not a type II-dual intersection formal context by Definition 7.*

**Theorem** **7.** *Let a formal context be (G,M,I). If (G,M,I) is a type II-dual intersection context, then $L(G,M,I^{c})$ and OEOL(G,M,I) are anti isomorphic.*

**Proof.**  Let $\eta :L_{E}\left(G,M,I^{c}\right)\to OEOL_{E}\left(G,M,I\right)$, for any $X\in L_{E}\left(G,M,I^{c}\right)$, $\eta \left(X\right)=X^{c}$. So let’s prove that it’s an anti isomorphic mapping. In fact, we know $L(G,M,I^{c})$ and $L_{o}\left(G,M,I\right)$ are anti isomorphic. And then through Theorem 4 and transitive properties of isomorphism, we can easily get η is an anti isomorphic mapping. □

## 4. The Characteristics of Formal Context Based on the Relationship among Kinds of Concept Lattices

Next, we mainly discuss the inverse proposition of the theorems in Section 3.

**Theorem** **8.** *Let a formal context be (G,M,I). If $L_{E}\left(G,M,I\right)\subseteq L_{E}\left(G,M,I^{c}\right)$, then (G,M,I) is a type II-dual intersection formal context.*

**Proof.**  For any a∈M, we get $a^{*}\in L_{E}\left(G,M,I\right)$ by the property of operators *. In addition, since $L_{E}\left(G,M,I\right)\subseteq L_{E}\left(G,M,I^{c}\right)$, we can obtain $a^{*}\in L_{E}\left(G,M,I^{c}\right)$. So we can have $\left(a^{*},a^{*\bar{*}}\right)\in L\left(G,M,I^{c}\right)$. In addition, then we get $a^{*}={⋂}_{a_{j}\in a^{*\bar{*}}}a_{j}^{\bar{*}}={⋂}_{a_{j}\in a^{*\bar{*}}}G\backslash a_{j}^{*}$ by Definition 1. Thus, we can get *a* is a type II-dual intersection attribute by Definition 6. By the arbitrariness of *a*, we get (G,M,I) is a type II-dual intersection formal context by Definition 7. □

**Theorem** **9.** *Let a formal context be (G,M,I). If $L_{oE}\left(G,M,I\right)\subseteq L_{oE}\left(G,M,I^{c}\right)$, then (G,M,I) is a type I-dual intersection formal context.*

**Proof.**  For any a∈M, we get $a^{◊}\in L_{oE}\left(G,M,I\right)$ by the property of operators *◊*. In addition, since $L_{oE}\left(G,M,I\right)\subseteq L_{oE}\left(G,M,I^{c}\right)$, we can obtain $a^{◊}\in L_{oE}\left(G,M,I^{c}\right)$. So we can get $\left(a^{◊},a^{◊\bar{□}}\right)\in L_{o}\left(G,M,I^{c}\right)$. In addition, $a^{◊}=\left\{g\in G|g^{*}\cap \left\{a\right\}\neq \emptyset \right\}=\left\{g\in G|a\in g^{*}\right\}=\left\{g\in G|gIa\right\}=a^{*}$. Similarly, we can get $a^{\bar{◊}}=a^{\bar{*}}$. $a^{*}=a^{◊}={⋃}_{a_{j}\in a^{◊\bar{□}}}a_{j}^{\bar{◊}}={⋃}_{a_{j}\in a^{◊\bar{□}}}a_{j}^{\bar{*}}={⋃}_{a_{j}\in a^{◊\bar{□}}}G\backslash a_{j}^{*}$ by Definition 1. So we get $G\backslash a^{*}={⋂}_{a_{j}\in a^{◊\bar{□}}}a_{j}^{*}$ by De Morgan’s law. And then we get (G,M,I) is a type I-dual intersection formal by the arbitrariness of *a* context. □

**Theorem** **10.** *If $L_{E}\left(G,M,I\right)=L_{E}\left(G,M,I^{c}\right)$, then (G,M,I) is both a type I-dual intersection formal context and a type II-dual intersection formal context.*

**Proof.**  Since $L_{E}\left(G,M,I\right)=L_{E}\left(G,M,I^{c}\right)$, we can get $L_{E}\left(G,M,I\right)\subseteq L_{E}\left(G,M,I^{c}\right)$ and $L_{E}\left(G,M,I^{c}\right)\subseteq L_{E}\left(G,M,I\right)$. When $L_{E}\left(G,M,I\right)\subseteq L_{E}\left(G,M,I^{c}\right)$, we can obtain (G,M,I) is a type II-dual intersection formal context by Theorem 8. In addition, When $L_{E}\left(G,M,I^{c}\right)\subseteq L_{E}\left(G,M,I\right)$, we can obtain $\left(G,M,I^{c}\right)$ is a type II-dual intersection formal context by Theorem 7. In addition, when $\left(G,M,I^{c}\right)$ is a type II-dual intersection formal context, we can get (G,M,I) is a type I-dual intersection formal context by Theorem 1. In summary, we get (G,M,I) is both a type I-dual intersection formal context and a type II-dual intersection formal context. □

Similarly to Theorem 10, we have the following conclusion which is obviously true by combining with Theorem 9.

**Theorem** **11.** *Let a formal context be (G,M,I). If $L_{oE}\left(G,M,I\right)=L_{oE}\left(G,M,I^{c}\right)$, then (G,M,I) is both a type I-dual intersection formal context and a type II-dual intersection formal context.*

Let a formal context be (G,M,I). If L(G,M,I) and $L_{o}\left(G,M,I\right)$ are anti-isomorphic, then (G,M,I) is a type I-dual intersection context. This is not necessarily true. See the following example for details.

**Figure 6:**
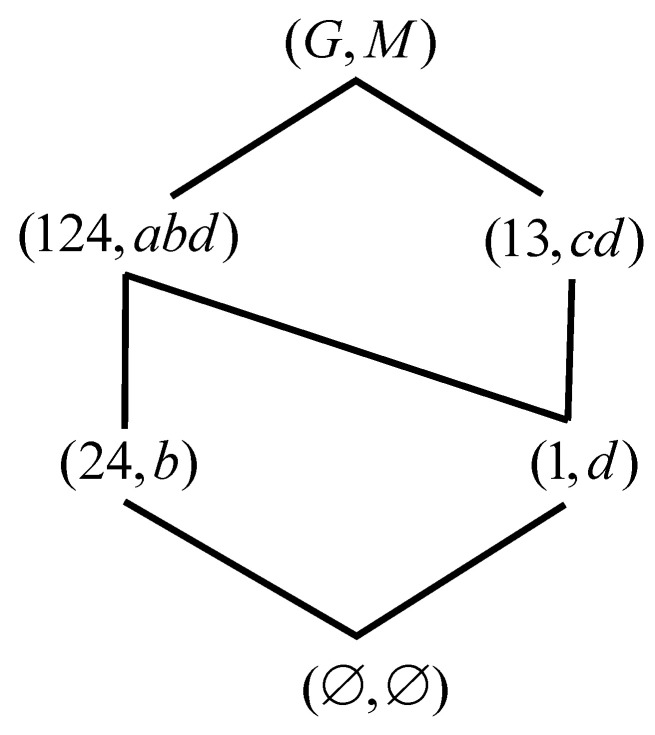
$L_{O}\left(G,M,I\right)$ of Table 3.

**Example** **7.** *The formal context (G,M,I) is shown in Table 3. Its the object oriented concept lattice is shown by Figure 6. We can easily obtain L(G,M,I) and $L_{o}\left(G,M,I\right)$ are anti-isomorphic. But according to Example 6, we know (G,M,I) is not a type I-dual intersection context and is not also a type II-dual intersection context.*

Next, let’s study the characteristics of formal context when two-way lattices and three-way lattice are isomorphic.

**Theorem** **12.** *Let a formal context be (G,M,I). If $L_{o}\left(G,M,I\right)$ and OEOL(G,M,I) are isomorphic, then (G,M,I) is a type II-dual intersection formal context.*

**Proof.**  Suppose $\left(X,A\right)\in L_{o}\left(G,M,I\right)$, we can get $X^{□}=A$, $A^{◊}=X$. In addition, $X^{\bar{□}\bar{◊}}\subseteq X$. So, $A^{◊}\cup X^{\bar{□}\bar{◊}}=X$, Thus, we can obtain $\left(X,\left(A,X^{\bar{□}}\right)\right)\in OEOL\left(G,M,I\right)$ by the definition of OEO-concept. Therefore, $L_{oE}\left(G,M,I\right)\subseteq OEOL_{E}\left(G,M,I\right)$. Similarly, we can get $L_{oE}\left(G,M,I^{c}\right)\subseteq OEOL_{E}\left(G,M,I\right)$. Since $L_{o}\left(G,M,I\right)$ and OEOL(G,M,I) are isomorphic, we can get $L_{oE}\left(G,M,I\right)=OEOL_{E}\left(G,M,I\right)$. Thus, $L_{oE}\left(G,M,I^{c}\right)\subseteq L_{oE}\left(G,M,I\right)$. Therefore, (G,M,I) is a type II-dual intersection formal context by Theorem 11. □

**Theorem** **13.** *Let a formal context be (G,M,I). If L(G,M,I) and OEOL(G,M,I) are anti-isomorphic, then (G,M,I) is a type I-dual intersection formal context.*

**Proof.**  Suppose (X,A)∈L(G,M,I), we can get $X^{*}=A$, $A^{*}=X$. In addition, $X\subseteq X^{\bar{*}\bar{*}}$. So, $A^{*}\cap X^{\bar{*}\bar{*}}=X$, Thus, we can obtain $\left(X,\left(A,X^{\bar{*}}\right)\right)\in OEL\left(G,M,I\right)$. So, $L_{E}\left(G,M,I\right)\subseteq OEL_{E}\left(G,M,I\right)$. Similarly, we can get $L_{E}\left(G,M,I^{c}\right)\subseteq OEL_{E}\left(G,M,I\right)$.

Suppose (X,(A,B))∈OEL(G,M,I), we define α:OEL(G,M,I)→OEOL(G,M,I), for any (X,(A,B))∈OEL(G,M,I), $\alpha \left(X,\left(A,B\right)\right)=\left(X^{c},\left(B,A\right)\right)$. So we can easily prove that it’s a reverse order isomorphic mapping. So, OEL(G,M,I) and OEOL(G,M,I) are anti isomorphic. In addition, we know L(G,M,I) and OEOL(G,M,I) are anti isomorphic. So we can obtain that L(G,M,I) and OEL(G,M,I) are isomorphic from the transitivity of isomorphism.

And we know $L_{E}\left(G,M,I\right)\subseteq OEL_{E}\left(G,M,I\right)$. Thus, $L_{E}\left(G,M,I\right)=OEL_{E}\left(G,M,I\right)$. Combining with $L_{E}\left(G,M,I^{c}\right)\subseteq OEL_{E}\left(G,M,I\right)$, we can get $L_{E}\left(G,M,I^{c}\right)\subseteq L_{E}\left(G,M,I\right)$. Therefore, $\left(G,M,I^{c}\right)$ is a type II-dual intersection formal context by Theorem 8. In addition, when $\left(G,M,I^{c}\right)$ is a type II-dual intersection formal context, we can get (G,M,I) is a type I-dual intersection formal context by Theorem 1. □

## 5. Conclusions

**Figure 7:**
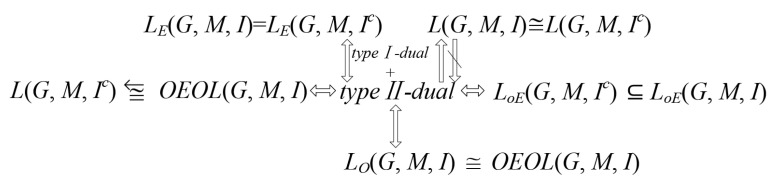
The detailed conclusions.

In the paper, we firstly gave a new attribute characteristic which is called type II-dual intersection attribute, and then we described the formal context and proposed the type II-dual intersection formal context. Secondly, we give the relation among some concept lattices based on the type II-dual intersection formal context and we proved some related theorems. Thirdly, the inverse proposition of related conclusions in Section 3 are studies. Some related theorems and counterexamples are give. The detailed conclusions are shown in Figure 7. However, there is a problem here that has not been solved. That is, what is the formal context like when it is both the type I-dual intersection formal context and the type II-dual intersection formal context. We conjecture that it is dual formal context whose every attribute has dual attribute. In the future, we will explore this issue.

## Figures and Tables

**Table 1:** A formal context (G,M,I).

*G*	*a*	*b*	*c*
1	×		
2		×	
3			×

**Table 2:** A formal context (G,M,I).

*G*	*a*	*b*	*c*	*d*	*e*
1	×	×		×	
2		×	×		×
3	×		×		×

**Table 3:** A formal context (G,M,I).

*G*	*a*	*b*	*c*	*d*
1	×		×	×
2	×	×		
3			×	
4	×	×		

## Data Availability

Not applicable.

## Abbreviations

The following abbreviations are used in this manuscript:

3WCA	Three-way concept analysis
FCA	formal concept analysis
(G,M,I)	three-way property oriented concept lattices
L(G,M,I)	the concept lattice
OEPL(G,M,I)	three-way property oriented concept lattices
$L_{o}\left(G,M,I\right)$	the object oriented concept lattices
$L_{oE}\left(G,M,I\right)$	the set of the object oriented concept extents
